# Single-Cell RNA-Seq Reveals the Cellular Diversity and Developmental Characteristics of the Retinas of an Infant and a Young Child

**DOI:** 10.3389/fcell.2022.803466

**Published:** 2022-03-21

**Authors:** Fangyuan Hu, Yuting Ma, Zaoxu Xu, Shenghai Zhang, Jiankang Li, Xinghuai Sun, Jihong Wu

**Affiliations:** ^1^ Eye Institute and Department of Ophthalmology, Eye and ENT Hospital, Fudan University, Shanghai, China; ^2^ NHC Key Laboratory of Myopia (Fudan University), Key Laboratory of Myopia, Chinese Academy of Medical Sciences, Shanghai, China; ^3^ Shanghai Key Laboratory of Visual Impairment and Restoration, Shanghai, China; ^4^ College of Life Sciences, University of Chinese Academy of Sciences, Beijing, China; ^5^ BGI-Shenzhen, Shenzhen, China

**Keywords:** human retinal cells, single-cell sequencing, cellular compositions, communication networks, inherited retinal diseases

## Abstract

The human retina, located in the innermost layer of the eye, plays a decisive role in visual perception. Dissecting the heterogeneity of retinal cells is essential for understanding the mechanism of visual development. Here, we performed single-cell RNA-seq to analyze 194,967 cells from the donors of infants and young children, resulting in 17 distinct clusters representing major cell types in the retina: rod photoreceptors (PRs), cone PRs, bipolar cells (BCs), horizontal cells (HCs), amacrine cells (ACs), retinal ganglion cells (RGCs), Müller glial cells (MGs), microglia, and astrocytes (ASTs). Through reclustering, we identified known subtypes of cone PRs as well as additional unreported subpopulations and corresponding markers in BCs. Additionally, we linked inherited retinal diseases (IRDs) to certain cell subtypes or subpopulations through enrichment analysis. We next constructed extensive intercellular communication networks and identified ligand-receptor interactions that play crucial roles in regulating neural cell development and immune homeostasis in the retina. Intriguingly, we found that the status and functions of PRs changed drastically between the young children and adult retina. Overall, our study offers the first retinal cell atlas in infants and young children dissecting the heterogeneity of the retina and identifying the key molecules in the developmental process, which provides an important resource that will pave the way for research on retinal development mechanisms and advancements in regenerative medicine concerning retinal biology.

## Introduction

The retina, composed of a complicated array of cell subpopulations, is the system responsible for light perception and visual signal transmission. In general, several retinal cell types compose the layered structure of the retina (Masland, 2012). The outer nuclear layer is made up of photoreceptors (PRs), including rod cells and cone cells, which are responsible for capturing photons and converting them into electrical signals that will later pass on to interneurons in the bipolar cell (BC) layer for further modulation. Furthermore, BCs transmit the processed signals to retinal ganglion cells (RGCs) via chemical synapses, and RGCs encode visual information as nerve impulses for transmission to the brain. During the full process of signal transduction, horizontal cells (HCs) cooperate with PRs to better integrate various light stimulation signals before passing them to BCs (Augustine, 1996; Masland, 2012), while amacrine cells (ACs) mediate the output of BCs through complex lateral regulation (Balasubramanian and Gan, 2014). Müller glial cells (MGs) are involved in extracellular environment regulation via nutrient intake, debris clearance, and structural and functional stabilization (Sarthy and Lam, 1978; Giannelli et al., 2011). Impairments in these cell types can lead to a range of retinal disorders, from minor visual defects to severe blindness.

In recent years, the rapid improvement of single-cell RNA sequencing (scRNA-seq) techniques has allowed us to study the retina in greater detail than before, revealing a tremendous number of subtypes within the traditionally defined classification. In human, 18 distinct cell clusters, including well-known retinal cells and novel rod cell subpopulations, have been identified from three post-mortem donors (Lukowski et al., 2019). Voigt et al. clinically and molecularly studied the retina of a 70-year-old patient with retinal degeneration attributed to autoimmune retinopathy (Voigt et al., 2020), thus providing evidence that glial cells have a distinct transcriptome in the context of human retinal degeneration; that study represented a complementary clinical and molecular investigation of a case of progressive retinal disease. Menon et al. constructed a single-cell transcriptomic atlas of the human retina and identified cell types associated with age-related macular degeneration (Menon et al., 2019). Hu et al. analyzed cells from the human fetal neural retina (NR) and retinal pigment epithelium (RPE) using scRNA-seq and revealed the tightly regulated spatiotemporal gene expression network of human retinal cells, capturing the key *in vivo* features of the development of the human NR and RPE and offering insightful clues for further functional studies (Hu H et al., 2019). Lu et al. performed scRNA-seq analysis at 16 time points of retinal development as well as four early stages of retinal organoid differentiation, identifying evolutionarily conserved patterns of gene expression during retinal progenitor maturation and specification of all seven major retinal cell types (Lu et al., 2020).

In this research, we analyzed 194,967 single-cell transcriptomic profiles from the infant and young child in different developmental stages. Unsupervised clustering defined 17 clusters, all of which were assigned to corresponding major retinal cell types. Communication network analysis connected the receptor-ligand pair with certain biological processes in the retina. Subsequently, we identified known subtypes of cone PRs as well as previously unknown subpopulations and corresponding markers in BCs by performing reclustering. By hypergeometric enrichment analysis, we further associated inherited retinal diseases (IRDs) with certain cell subtypes or subpopulations. Next, the differences between the retinas of the young child and adult were investigated. Intriguingly, we found that the status and functions of PRs changed between the retinas of the young child and adult. Our study provides a valuable data resource for retinal development research and the advancement of retinal regenerative medicine.

## Materials and Methods

### Ethics Statement

The collection of human retinas was approved by the Ethics Committee of the Eye and ENT Hospital of Fudan University (2019025) and in accordance with the Code of Ethics of the World Medical Association (Declaration of Helsinki) for medical research involving human subjects. All protocols were performed according to the “Interim Measures for the Administration of Human Genetic Resources” administered by the Chinese Ministry of Health. Written informed consent was obtained from the minors’ next of kin for the publication of any potentially identifiable images or data included in this article.

### Human Retina Collection

The retinal tissues came from a 10-month-old (TenM) and a 2-year-old (TwoY) postmortem human donors. The donors’ eyeballs were collected by Eye and ENT Hospital of Fudan University for corneal transplantation, and eyeball samples were acquired within 12 h after the death of the donors. The remaining eyeball tissues were used to extract retinal tissues. The retinal tissues were exposed by removing the iris, lens, and vitreous successively. Both whole retinas from each donor were carefully dissociated and placed in precooled HBSS (Gibco, Thermo Fisher Scientific, Waltham, MA, United States) without a retinal pigment epithelium (RPE)/choroid layer. The whole retina from one of the eyeballs of the two-year-old donor was digested into a single-cell suspension in trypsin-EDTA solution (Gibco, Thermo Fisher Scientific, Waltham, MA, United States) (containing 0.25% trypsin and 0.02% EDTA) at 37°C for 20 min, and the remaining retinal tissues were immediately frozen in liquid nitrogen. Then, the single-cell suspension was filtered through a 40 μm cell strainer (BD, Franklin Lakes, New Jersey, U.S.) to eliminate any clumped cells. The freshly prepared cell suspension was immediately used for the construction of single-cell sequencing libraries.

### Single-Cell cDNA Library Preparation and High-Throughput Sequencing

The freshly prepared cell suspensions were used to generate cDNA libraries with a Single Cell 3′ Library and Gel Bead Kit V2 (10×Genomics, Pleasanton, California, United States) according to the manufacturer’s instructions. Flash-frozen tissues were homogenized in 2 ml ice-cold lysis buffer (10 mM Tris-HCl, pH 7.4/10 mM NaCl, 3 mM MgCl_2_, 0.1% NP40, and protease inhibitors) and then homogenized on ice in an RNase-free 2 ml glass Dounce homogenizer (Sigma, St. Louis, Missouri, United States) 15× with a loose pestle and 15× with a tight pestle. The homogenate was passed through a 40 µm filter to remove the block mass. The cell filtrate was subjected to density gradient centrifugation. Then, 400 µL of cell filtrate was mixed with 400 µL of 50% iodixanol solution (Sigma, St. Louis, Missouri, United States) in 2 ml lo-Bind tubes (Sigma, St. Louis, Missouri, United States). After 29% iodixanol solution and 35% iodixanol solution were carefully layered into the bottom, the tube was centrifuged at 3,000 × *g* at 4°C for 30 min.

Nuclei were resuspended in ice-cold 1× PBS (Gibco, Thermo Fisher Scientific, Waltham, MA, United States) containing 0.04% BSA (Sangon, Shanghai, China) and centrifuged at 500 × g for 5 min. The supernatant was discarded, and then 50 μL ice-cold 1 × PBS containing 0.04% BSA and 0.2 U/µl RNase Inhibitor (NEB, Ipswich, Massachusetts, United States) was combined with the cell pellet by gentle pipetting using a regular-bore pipette tip. The nuclear concentration was determined using a hemocytometer (QIUJING, Shanghai, China). Next, the Nuclei were loaded on a Chromium Single Cell Controller (10× Genomics, Pleasanton, California, United States) to generate single-cell gel beads in emulsion (GEMs) by using a Single Cell 3′ Library and Gel Bead Kit V2. Captured cells released RNA and barcoded in individual GEMs. Libraries were generated from each donor sample according to the manufacturer’s instructions. Indexed libraries were converted with an MGIEasy Lib Trans Kit (MGI, Shenzhen, China) and then sequenced on the MGISEQ 2000 (MGI, Shenzhen, China) platform with paired-end 26 bp+100 bp+8 bp (PE26 + 100+8).

### Preprocessing and Quality Control of scRNA-Seq Data

We first used Cell Ranger 3.0.2 (10×Genomics) to process raw sequencing data, and then Seurat (Satija et al., 2015) was applied for downstream analysis. Before we started downstream analysis, we focused on four filtering metrics to guarantee the reliability of our data. 1) We filtered out genes that were detected in less than 0.1% of the total cell number to guarantee the reliability of each gene. 2) We filtered out cells whose percentage of expressed mitochondrial genes was greater than 10%. 3) We also filtered out cells whose unique molecular identifier (UMI) counts were either less than 1.5 offset to the first quantile or greater than 1.5 offset to the third quantile of total UMI counts to filter out doublet-like cells. 4) We filtered out cells with fewer than 200 detected genes.

Data quality control is carried out as follows:

1) Filtering Low-quality genes.

Genes that are expressed in insufficient cells contribute little to the analysis of cell types and population, resulting in low capture sensitivity, low expression quantity, and low reliability, so these genes should be filtered.

2) Filtering low quality cells.

a. High proportion of mitochondrial genes.

If the proportion of mitochondrial genes is high and the cell types are not a particularly mitochondria-rich cell, it indicates those cells capture insufficient genes and should be removed. The reason for the high proportion of mitochondrial genes could be that the cell activity is weak and tends to apoptosis, or cell membrane is damaged, which leads to mRNA dissociation and less mRNA is captured.

b. too low or too high total gene number and counts.

During library preparation, RNA may be lost due to cell lysis or low efficiency of cDNA capture and amplification. Cells with a low gene number and counts are considered low quality cells and should be removed. Also, cells with too high gene number or counts may indicate doublet cells (capture two cells as one) and therefore should be filtered.

### Identification of Cell Types and Subtypes by Dimensional Reduction

Seurat is the current mainstream analysis tool for single-cell data. The heterogeneity of the retina was determined using the Seurat R package (Satija et al., 2015). We performed Seurat alignment to eliminate batch effects, allowing us to combine data from multiple samples. Then, we used the function JackStrawPlot, which comes with the Seurat package, to determine the significant PC dimension to use for the downstream clustering analysis. The function JackStrawPlot is mainly used for PC significance calculation. The top twelve PCs were used for cluster identification with a resolution of 1.0 using the k-nearest neighbor (KNN) algorithm and visualization using the uniform manifold approximation and projection (UMAP) algorithm. Cell types were assigned by the expression of known cell-type markers and functional enrichment analysis. FindAllMarkers is a function for difference testing of expression data in Seurat, the default is Wilcoxon Rank Sum test. The FindAllMarkers was used to identify marker genes for each cluster using the default parameters.

### Functional Enrichment Analysis

The functional enrichment analysis included two parts: Gene Ontology (GO) terms and KEGG pathways. Lists of genes were analyzed using the clusterProfiler R package, and the Benjamini-Hochberg (BH) method was used to correct for multiple comparisons. GO terms with a *p* value less than 0.01 and KEGG terms with a *p* value less than 0.05 were considered significantly enriched.

### Construction of a Cellular Communication Network

The cellular communication network was constructed by utilizing CellPhoneDB database (Efremova et al., 2020). The significant cell-cell interactions were selected with *p*-value < 0.05. The parameter “--threshold” was set to 0.01, which indicates that Ligands/Receptors encoding genes were considered to be expressed by the cell type with higher than 1% cell expressing the gene in the corresponding cell types.

### Construction of Intercellular Correlation Network

To reduce noise, we averaged the expression of every 100 cells within the cluster and then calculated the pairwise Pearson correlation coefficients between pairs of dots based on their average expression profiles. Interdot relationships were shown if their Pearson correlation was greater than 0.8. Visualization of the correlation network was achieved using Cytoscape (Shannon et al., 2003).

### Construction of Pseudotime Trajectory Using Variable Genes

Monocle is currently the mainstream tool for pseudotime analysis, which is to construct the change trajectory between cells by sorting the information of cell transcript abundance, so as to reshape the change process of cells over time. Monocle can be used for the simulation of cell differentiation lineages during development and cytopathic trajectories in disease progression. Also, the Monocle2 R package (version 2.10.1) was performed to construct single-cell pseudotime trajectories to identify developmental transitions (Trapnell et al., 2014; Qiu et al., 2017a; Qiu et al., 2017b). We used highly variable genes identified by the “estimateDispersions” function to sort cells in pseudotime order. “DDRTree” was applied to reduce the dimensional space, and the minimum spanning tree of the cells was plotted by the visualization function “plot_complex_cell_trajectory”. Branch expression analysis modeling (BEAM) tests were performed on the first branch points of the cell lineage using all default parameters. The “plot_genes_branched_pseudotime” function was used to plot two genes for each lineage.

### Branch Expression Analysis Modeling

Monocle uses BEAM to calculate the changes in gene expression as cells pass from an early developmental stage through the branch and to identify the DEGs between different branches. We used the “BEAM” function with default parameter settings, and genes with a *p* value less than 0.01 were considered to be significant.

### Regulatory Network Construction

To construct regulatory networks, we first downloaded the *Homo sapiens* TF list from AnimalTFDB 3.0 (Hu Y et al., 2019) as a TF reference and extracted TFs from the marker gene list of each cluster. The extracted TFs were used as input of the function “GENIE3” in the GENIE3 R package (Huynh-Thu et al., 2010) with no predefined target. A value of 0.6 was used as the threshold above which an interaction was considered to be significant. The network was plotted using Cytoscape (Shannon et al., 2003).

### Disease Enrichment Analysis

For disease enrichment analysis, we first retrieved retinal disease risk genes from the RetNet database (https://sph.uth.edu/retnet/disease.htm) and then performed the hypergeometric test (“dhyper” function in R) using the risk genes and DEGs of each cluster. A value of 0.05 was used as a threshold to define significance.

## Results

### Sample Preparation and Preliminary Data Validation

**FIGURE 1:**
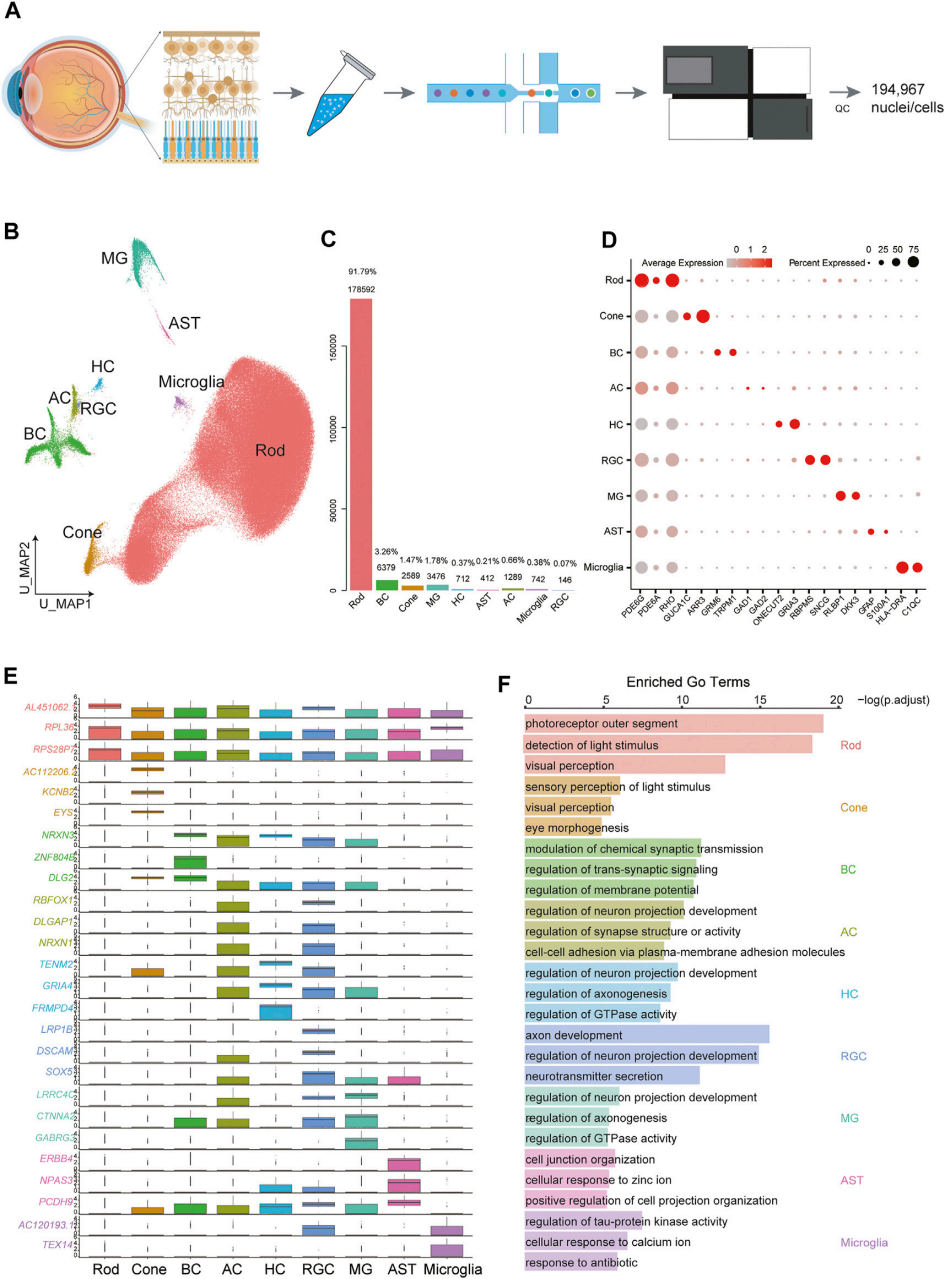
Cellular diversity in the retina. **(A)** Scheme of the experimental and bioinformatic design of our study. **(B)** A UMAP map of all libraries. Cells are colored by cell-type cluster. **(C)** Barplot showing the percentage and number of each cell type in the whole dataset. **(D)** Dot plot for markers of cell types. The size of each dot represents the percentage of cells in each cluster. The gradient from gray to red indicates a gradient from low to high gene expression. **(E)** Boxplot showing the top differentially expressed gene for each of the 9 cell types. **(F)** Selected GO terms enriched in each cellular cluster. Bar length denotes the adjusted *p* value and color denotes the cell type.

We collected both whole retinas from each donor (a 10-month-old infant and a 2-year-old young child, hereafter termed TenM and TwoY, respectively) and dissociated them into single-cell or single-nucleus suspensions without surface marker preselection (Figure 1A). For each donor, we constructed three separate single-cell libraries, which were subsequently sequenced on different lanes. As shown in the table of Supplementary Figure S1A, the libraries TenM4, M5, and MZ were derived from the TenM retinas (same donor), and all of these were single-nucleus RNA (snRNA) libraries. Libraries TwoY6, Y8, and YZJ were derived from the TwoY retinas (same donor). Among them, TwoY6 and Y8 were snRNA libraries, and TwoYZJ was scRNA library. After filtering, a total of 194,967 nuclei/cells were retained for subsequent analysis. The average number of genes per cell in each library was between 316 and 680, and the average number of reads in a single cell ranged from 8,001 to 128,753 (Supplementary Figure S1A,B). All the samples correlated quite well with each other with Spearman correlation efficiency ranging from 0.68 to 1.00 (Supplementary Figure S1C). Samples at the same time point showed stronger correlation than the samples at different time points (higher than 0.75).

### Global Cellular Heterogeneity in the Retina

We set out to create a map of the human retina. Overall, 194,967 cells were clustered into 17 clusters (Supplementary Figure S1D), and we were able to identify several major retinal cell types and glial cells (Figure 1B): rod PRs (C0-C5, C10, C15), cone PRs (C8), BCs (C6, C9), HCs (C13), ACs (C11_1), RGCs (C11_2), MGs (C7), microglia (C12), and astrocytes (ASTs) (C14). Rod PRs expressed high levels of *PDE6G* (Lagman et al., 2016) and *RHO* (Lukowski et al., 2019)*,* and cone PRs were characterized by the expression of *ARR3* and *GUCA1C* (Lukowski et al., 2019). BCs exhibited specific expression of *GRM6* (Ueda et al., 1997). To assess the cellular composition of the retina, we calculated the percentage of each cluster in the whole dataset (Figure 1C). Rod PRs accounted for the majority (91.79%) of the whole dataset, while cones composed a very small portion (1.47%). The second most common cell type was BC (3.26%). We performed differentially expressed genes (DEGs) and functional enrichment analysis based on canonical markers (Figures 1D,E, Supplementary Table S1). Additionally, function-related GO terms were enriched in the corresponding clusters (Figure 1F, Supplementary Table S2). For example, PR clusters were strongly associated with sensory perception of light stimuli and visual perception, while BCs were enriched in GO terms related to modulation of chemical synaptic transmission. RGCs are the innermost neurons of the retina, and their protruding axons form the optic nerve; these cells displayed enrichment in axon development, regulation of neuron projection development and neurotransmitter secretion.

We noticed that cells from different libraries mix very well, suggesting little batch effects (Supplementary Figure S1D).

More importantly, we compared the cell populations of the 10-month and 2-year-old samples and found that the number distribution of microglial populations differed significantly in the two samples of different ages. As shown in Supplementary Figure S1D, the number of microglial populations in the two-year-old samples was significantly higher than that in the 10-month-old samples. Microglia are the predominant immune cell types and also the key population of glial cells in the retina. We performed DEGs analysis on the microglia of the 10-month-old and two-year-old samples, and the results showed that the inflammation and immune regulation-related factors (CXCL8, CXCL16, CCL3, CCL3L1, etc.) were significantly expressed in the microglia of the two-year-old samples (Supplementary Figure S1E). This suggested that microglia in the retina of early childhood were fully developed and considered to be possess mature immunomodulatory function compared to infancy.

**FIGURE 2:**
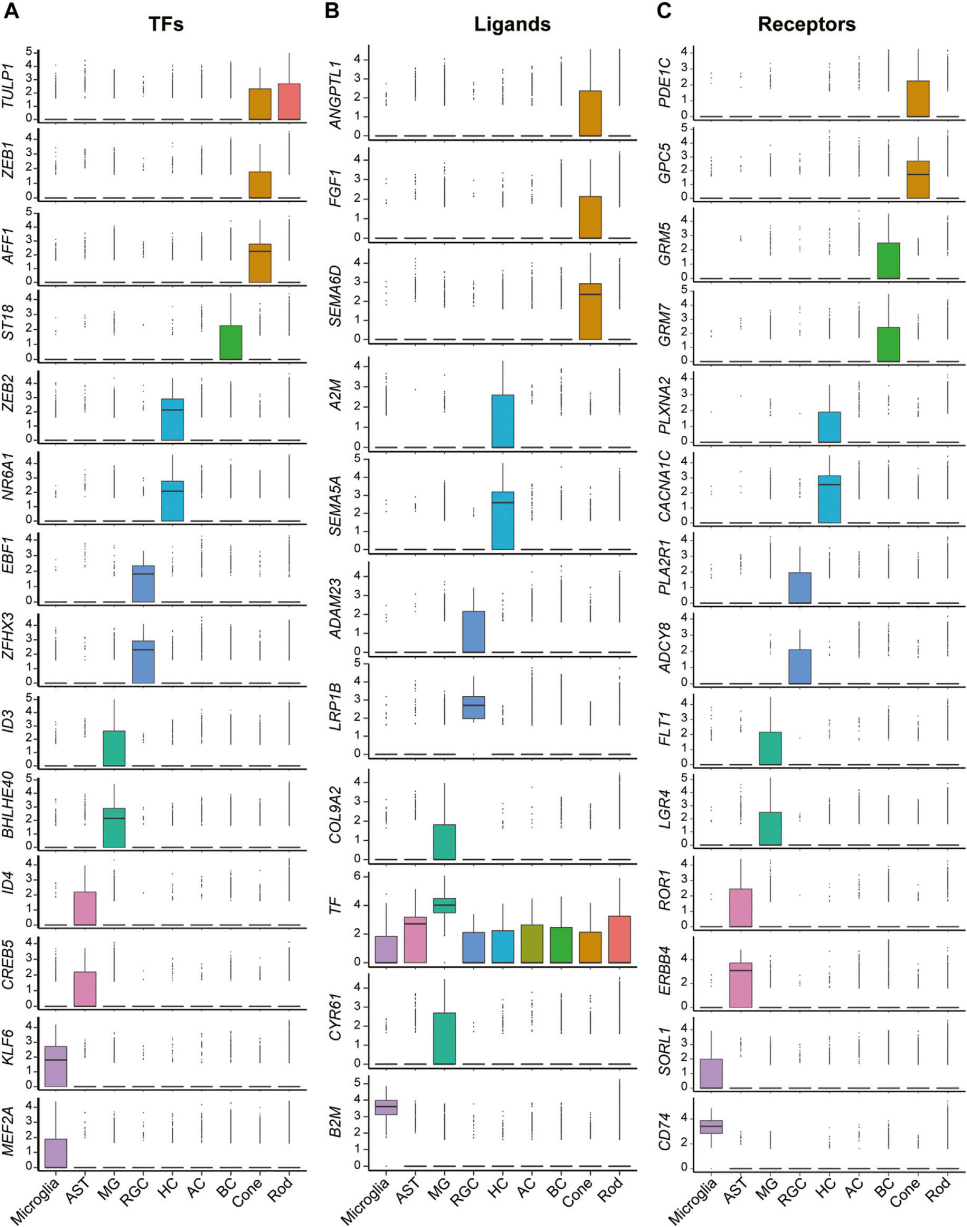
Expression patterns of differentially expressed functional genes of cells in retinas. **(A)** Boxplots show the expression patterns of differentially expressed transcription factor encoding genes of each cell clusters. **(B)** Boxplots show the expression patterns of differentially expressed ligand encoding genes of each cell clusters. **(C)** Boxplots show the expression patterns of differentially expressed receptor encoding genes of each cell clusters.

In addition to those well-known canonical markers of each cell type, we also found a variety of transcription factors (Figure 2A), ligands (Figure 2B), and receptors (Figure 2C) specifically expressed in each cell type. For example, *ZEB1*, a zinc finger transcription factor-encoding gene that may play an important role in the transcriptional repression of interleukin 2 (Wang et al., 2009), was expressed exclusively in the cones. *ST18*, another zinc finger transcription factor-encoding gene related to primary angle-closure glaucoma (Wei et al., 2014) and breast cancer (Yang et al., 2008), was found to be highly expressed in the BCs. *ZEB2* was found specifically expressed in the HCs. The protein encoded by *ZEB2* is related to TGF-beta receptor signaling and TGF-beta signaling pathways (Zhou et al., 2018). In addition, other transcription factors such as *EBF1*, *BHLHE40*, *CREB5*, and *KLF6*, were exclusively expressed in RGCs, MGs, ASTs, and microglia, respectively. Cellular surface receptor–encoding genes such as *SEMA6D*, *SEMA5A*, *LRP1B*, *TF*, and *B2M* were expressed especially strongly in cones, HCs, RGCs, MGs, and microglia, respectively. Ligand-encoding genes such as *GPC5*, *CD74*, *ERBB4*, *FLT1*, *PLA2R1*, *CACNA1C*, and *GRM5* showed exclusive expression patterns in cones, BCs, HCs, RGCs, MGs, ASTs, and microglia, respectively.

### Functional Subpopulations Among Cones and BCs

Due to the high heterogeneity of retinal cells, our next step was to provide a more specific map for major cell types in the retina. Cones and BCs are two types of cells that are primarily involved in signal transduction in the retina; as a result, we extracted clusters corresponding to these two cell types and performed reclustering analysis to further investigate heterogeneity.

**FIGURE 3:**
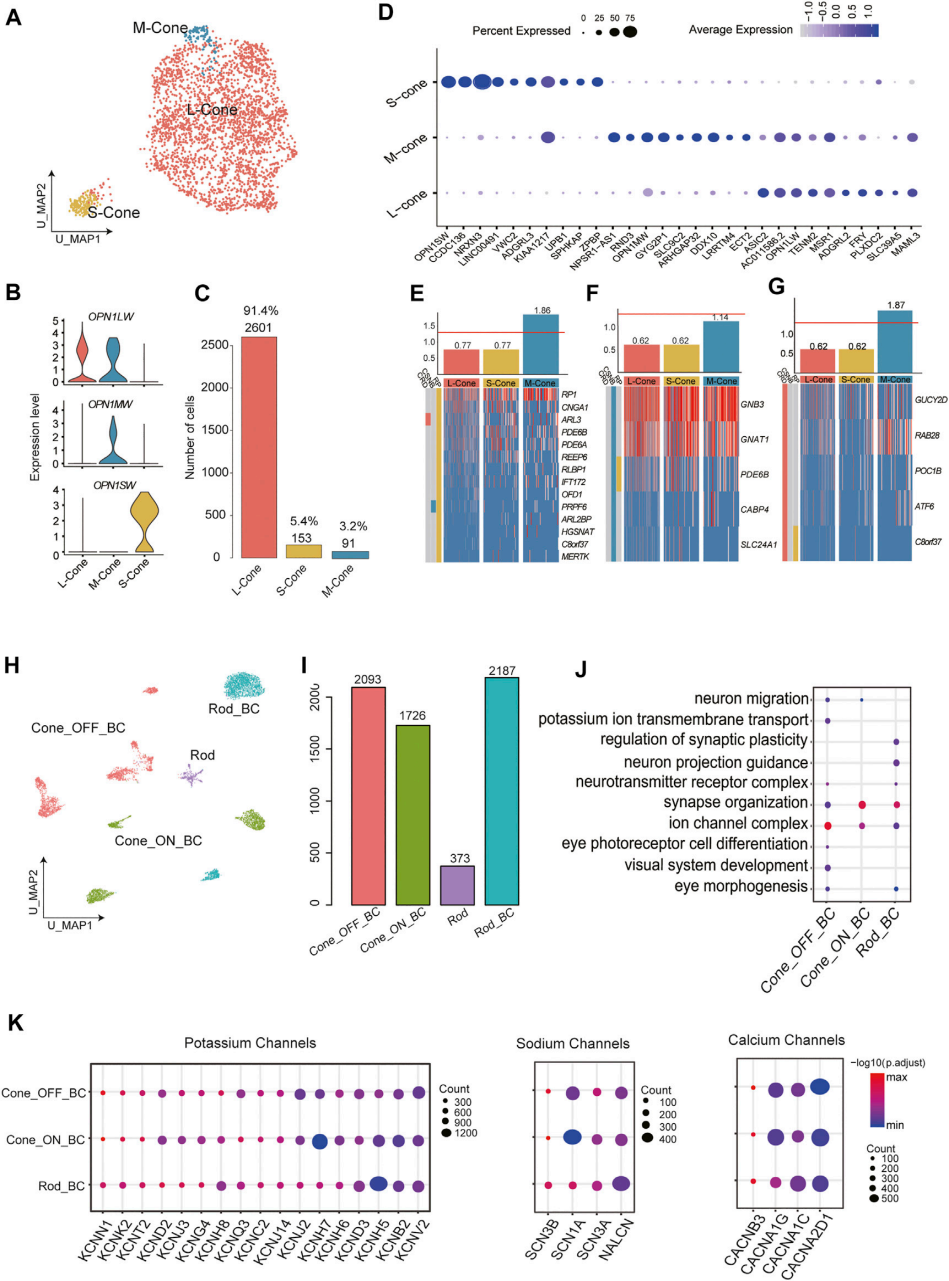
Cellular diversity within cone PRs and BCs. **(A)** A UMAP of cone PRs single-cell data. Cells are colored by cluster (subtypes). **(B)** Annotation of cone subclusters: expression of three opsins (OPN1SW, OPN1MW, and OPN1LW) in three cone subclusters **(C)** Barplot showing the number of each cell type in cone subclusters. **(D)** Bubble plot showing the expression of differentially expressed genes in each cluster. Dot size represents the percentage of cells expressed corresponding gene and color represents the expression level. **(E)** Cone subtype enrichment of RP, **(F)** CSNB, and **(G)** CRD risk genes, respectively. Heatmap: expression of corresponding disease risk genes in cell subtypes. Cells are ordered by cluster. Bar graphs: numbers indicate log2 *p* value. The red line indicates significance. **(H)** A UMAP map of BCs single-cell data. Cells are colored by cluster (subtypes). **(I)** Barplot showing the number of each cell type in the whole dataset of bipolar subtypes. **(J)** Enriched GO terms in BC subclusters. Dot size denotes the gene ratio and color denotes the adjusted *p* value. **(K)** Bubble plot showing specific expression of Potassium, Sodium, and Calcium channels encoding genes in BC subclusters. Dot size represents the percentage of cells expressed corresponding gene and color represents the expression level.

Based on previous studies, the human retina contains three types of cone PRs that can be differentiated by the expression of three opsins (*OPN1SW, OPN1MW, OPN1LW*); these cone types are responsible for the perception of colors with different wavelengths (Neitz and Neitz, 2011). To assess the heterogeneity of cones, we performed reclustering in cone PR clusters and divided them into 3 subclusters. Although *OPN1MW* and *OPN1LW* have highly homologous sequences, we distinguished the S-cones from M-cones and L-cones using opsin-encoding genes (Figures 3A,B). In this cone PR dataset (2845 cone PRs), L-cones constituted 91.4% of the total cells, and the remaining were S-cones and M-cones (Figure 3C). Next, we conducted differential gene expression analysis to reveal new genetic characteristics of S-cones, M-cones, and L-cones (Supplementary Table S3). Genes highly and specifically expressed by S-cones (e.g., *OPN1SW, CCDC136, NRXN3*) and M-cones (e.g., *RND3, SLC9C2, APHGAP32*) were identified (Figure 3D), providing further molecular evidence for future cone PR studies.

BCs, which serve as interneurons, receive electrical impulses from PRs and then transmit them to RGCs after processing the signals in various manners. The diverse subtypes of BCs fill a variety of functional roles. Previous research using ∼25,000 BCs has experimentally confirmed 15 subtypes of BCs (Shekhar et al., 2016). BCs can be divided into two categories according to the type of PR to which they connect. Another taxonomy classifies BCs into ON and OFF types based on whether they are excited or suppressed by increasing illumination. In our BC dataset (6,379 BCs), unsupervised reclustering produced 11 distinct subclusters (Supplementary Figure S2A). Importantly, C6 were thought to be PR contamination and excluded from downstream analysis due to the modest expression of BC markers and the enrichment of GO terms concerning light perception among the DEGs of C6. C0 and C5 were annotated as rod bipolar cells (RBCs), showing high expression of PRKCA. According to the clear expression patterns of GRM6 and GRIK1, C2, C4, C7, C9, and C10 were considered as OFF BCs, while the remaining C1, C3, and C8 were ON BCs (Figure 3H, Supplementary Figure S2B).

Moreover, we counted the total numbers of cells in different BC subtypes (Figure 3I). We further explored the function of DEGs by functional enrichment analysis (Figure 3J, Supplementary Table S3). GO terms that are closely related to signal transduction (e.g., modulation of chemical synaptic transmission) and neuron recognition and morphogenesis (e.g., axon guidance and synapse organization) were enriched in almost all clusters to varying extents. Next, we sought to fully describe the type-specific functional roles of BC subtypes by assessing the expression of sodium, calcium, and potassium ion channel subunits (Figure 3K), which reflects the regulatory effect of ion channels on the transmission of neural signals. BK channels, which are responsible for the fundamental control of neural excitability (Laumonnier et al., 2006) were extensively expressed in BC subpopulations. Genes encoding sodium channel subunits were expressed selectively among subtypes. In addition to *SCN1A, SCN2A, SCN3A*, and *SCN8A*, which were previously reported to be expressed in cone bipolar cells (CBCs) in goldfish (Zenisek et al., 2001), we noticed that *SCN3A* and *SCN3B* also showed specific expression among RBCs. Genes encoding the calcium channel alpha and delta subunits also showed varying expression levels among subtypes.

### Enrichment Analysis of Disease-Causing Genes Revealed Critical Cell Subpopulations Related to RP, CSNB and CRD

We retrieved retinal disease–causing genes from the RetNet database (https://sph.uth.edu/retnet/disease.htm) and performed a hypergeometric test to determine whether these disease-related genes were enriched among the DEGs of certain clusters, aiming to discover putative disease-associated cell types and thus provide new insights for retinal diseases. Most of the disease types we investigated in this study are caused by malfunctions of PRs. Retinitis pigmentosa (RP), whose symptoms generally include trouble seeing at night and decreased peripheral vision, is characterized by retinal cell degeneration, beginning with rods and then progressing to cones (Berson, 1996). Consistent with these findings, enrichment of RP-related genes occurred in M-cones (Figure 3E). This indicated that M-cones might be related to the pathogenesis of RP, and the corresponding markers we identified might be helpful in defining the scope of target cells for therapies. Congenital stationary night blindness (CSNB) is a rare nonprogressive retinal disorder, which are caused by defects localized to PR synapses (Liu et al., 2013). As shown in Figure 3F, three subtypes of cone PRs were not significantly associated with CSNB. Cone-rod dystrophy (CRD) is a series of relevant eye disorders causing vision loss, which becomes more severe over time. Unlike RP, CRD is characterized by an initial loss of color vision and visual acuity due to loss of cone function, followed by rod cell deterioration (Gill et al., 2019). M-cones displayed close connections with CRD occurrence, showing significant enrichment [−log(*p* value)] over 1.5 (Figure 3G). Among cone PRs, S-cones seemed to have no significant connection with any retinal disease we examined, while M-cones were the major cone PR subtype causing or being affected by retinal diseases (Figures 3E–G).

### Intercellular Correlation Network of Heterogeneity in the Retina

**FIGURE 4:**
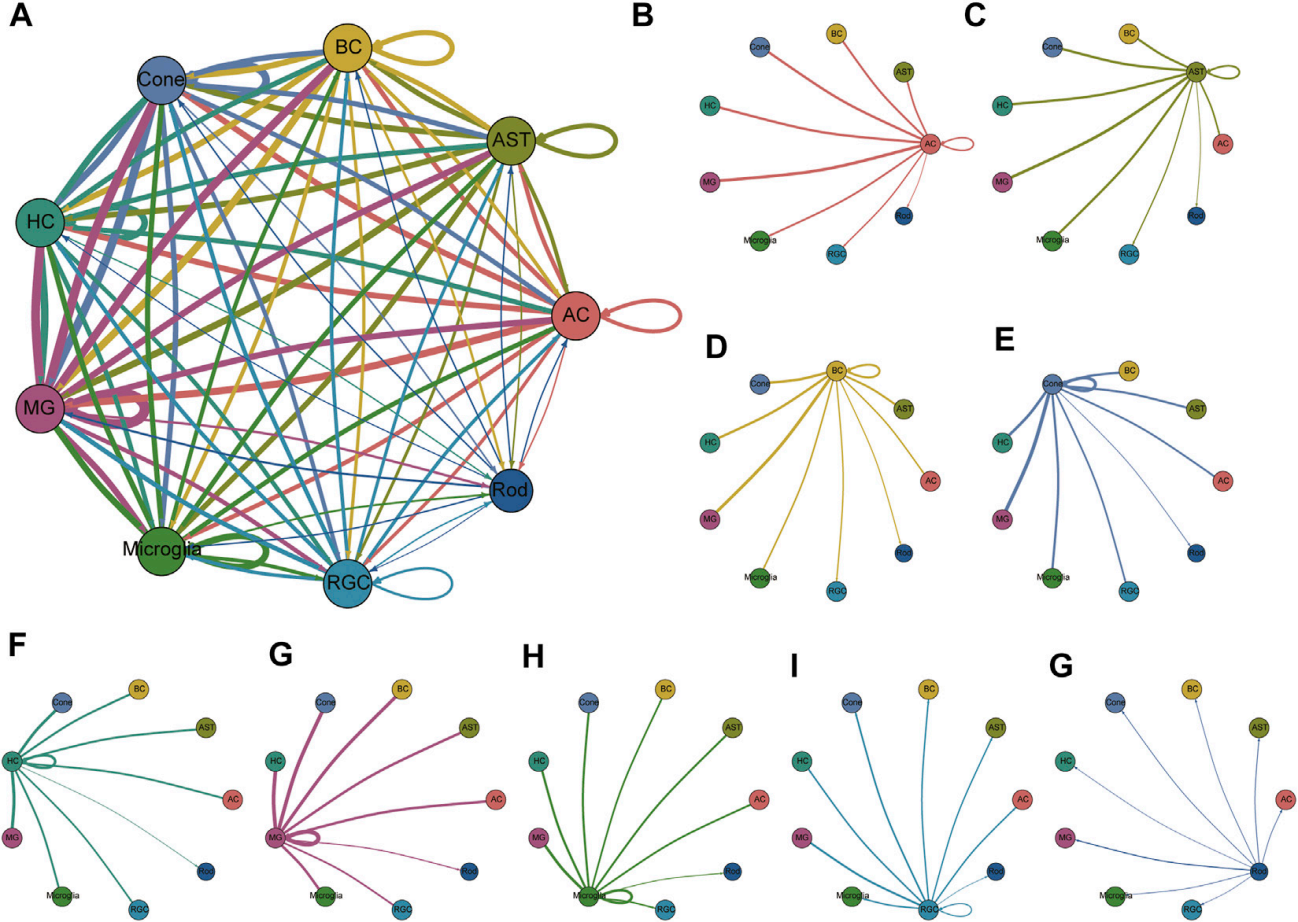
Communication network of all clusters and communication networks separated by clusters. **(A)** Overall cellular communication network. Nodes with different colors represent different cell types, and lines represent potential receptor-ligand interactions between the two cell types. The line thickness is proportional to the number of ligands where cognate receptors are present in the recipient cell population, and it can be regarded as the interaction strength. Lines with fewer than 1 interaction are not shown. The specific receptor-ligand pair information is shown in Supplementary Table S4. **(B–G)** The interactions of each cell type with the other cell types are shown separately.

Next, we explored the intercellular communication network within the retina. The cellular interactions were inferred using published ligand-receptor pairs. The expression patterns of ligand-receptor pairs in the networks revealed dense intercellular communication networks, especially in glia and BCs (Figure 4, Supplementary Table S4). Nevertheless, we observed that rod PRs interacted more frequently with other retinal cell types than with PR subpopulations, as rod PR subpopulations exhibited a relatively sparse network, showing <1 interaction among subpopulations within themselves and a few with other cell types. The most frequent interaction that appeared in our dataset was RTN4—LINGO1, which has been reported to participate in regulating the radial migration of cortical neurons in the mouse brain (Mathis et al., 2010), implying that RTN4—LINGO1 might be involved in the migration of neural cells in the retina. The combination of NRG3 and ERBB4 was also extensively expressed in retinal neural cell—MG interactions. NRG3 is a member of the neuregulin gene family. As the ligand of the ERBB transmembrane tyrosine kinase receptor family, NRG3 binds to ERBB4 and induces the phosphorylation of tyrosine. NRG3 has been reported to play a key role in regulating the growth and differentiation of glial cells, and its signal transduction role *via* ERBB4 is essential for neural circuit formation (Mei and Nave, 2014). SEMA3A, specifically expressed in rod PRs (C0, C16), can bind to PLXNA4 and engage in multiple biological actions, such as axon pruning and dendrite branching. In particular, HLA-A was highly expressed in MGs, and its cognate receptor APLP2 was detected in the majority of clusters, covering PRs, BCs, and MGs. HLA-A is a member of the human-specific major histocompatibility complex (MHC) antigen family, and its interaction with APLP2 is able to enhance the endocytosis and antigen-presenting ability of immune cells. Moreover, PTN ligands were frequently present in MGs. PTN is an important regulator of embryonic development of the nervous system and the vascular system. Several studies have confirmed that PTN is involved in the regulation of neovascularization and may also be involved in nerve cell repair.

### Comparison of the Retinas in Young Children and Adults

**FIGURE 5:**
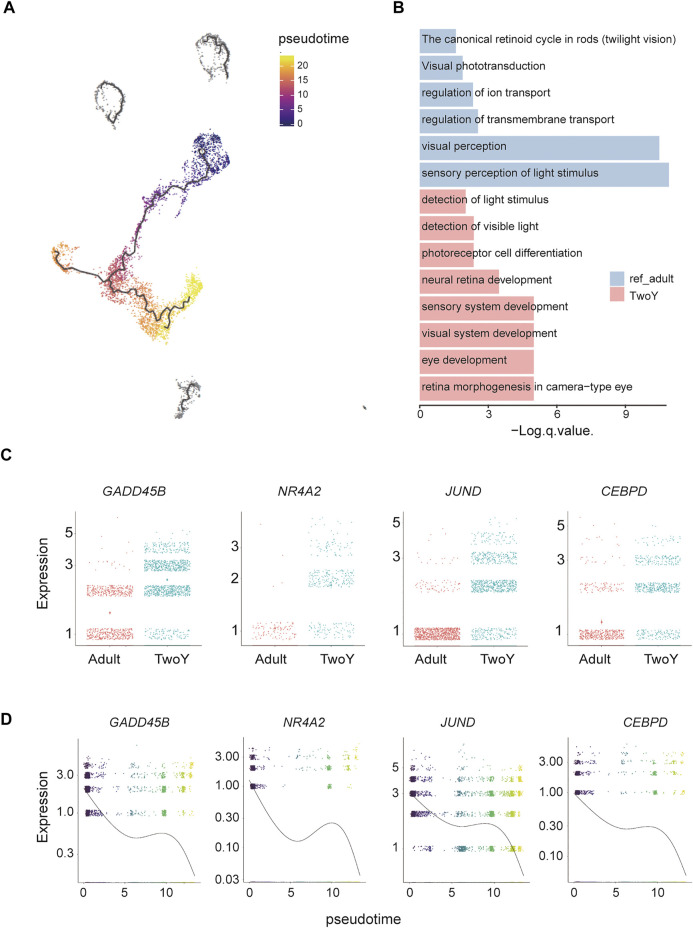
Trajectory analysis and comparison with reference adult data of MGs. **(A)** Cell lineage relationships of reference of adult cells and MGs. Monocle recovers a branched single-cell trajectory. Each dot represents a single cell. The dark colors are the starting point of the trajectory, which represent the cells in early development stage, mainly derived from TwoY MGs; the light colors represent the end of the trajectory, which represent late developmental cells, mainly derived from adult MGs. The dark gray clusters (top part of the UMAP map) are cell populations that do not conform to the developmental trajectory. **(B)** The enriched GO terms show the different cellular properties of the MGs in adults and young children. **(C)** Comparison of reference data from the MGs populations of adults and young children by differential gene expression. **(D)** Illustration of the decrease in related genes during evolution in a pseudotime analysis by Monocle.

We mixed TwoY retinal data (standard scRNA database) and adult retinal data from a previous study (Lukowski et al., 2019) and then conducted GO term enrichment analysis to study the upregulated/downregulated genes in young children and adults. MGs are one of the most common types of glial cells in the retina and play a role in maintaining the stability of retinal cell structure and function. It has been reported that MGs show similar properties to retinal precursor cells in human and mouse cell cultures and can differentiate into photoreceptors (Giannelli et al., 2011; Singhal et al., 2012). To further investigate the developmental relationship of the MG cluster, we used Monocle to reconstruct the pseudotime trajectory. As shown in Figure 5A, dark to light colors reflected the developmental trajectories of MGs, and the distribution of cells from young children to adults suggested that the trajectories conformed to biological developmental characteristics. Compared with those of adults, the functional GO terms of MGs in young children were enriched in the development of sensory and visual systems, while adults showed more mature MG properties, such as the regulation of transmembrane transport and ion transport (Figure 5B). In addition, we found that *GADD45B*, *NR4A2*, *JUND*, and *CEBPD* were highly expressed in MGs from young children (Figure 5C), and the pseudotime analysis also indicated that these four genes decreased over time (Figure 5D). More importantly, these four genes are closely related to cell development and differentiation. It has been reported that the expression of *GADD45B* is increased upon embryonic stem cells differentiation (Zhang et al., 2021). Meanwhile, *GADD45B* is considered as a myeloid differentiation primary response gene (Abdollahi et al., 1991). *NR4A2*, also known as nuclear receptor *NURR1*, plays a critical role in the differentiation and maintenance of dopaminergic neurons (Beiki et al., 2021). Activator Protein-1 family member *JUND* is involved in regulating the transcriptional expression of genes related to cell differentiation (Shaulian et al., 2000). Moreover, the main function of *CEBPD* is the regulation of critical cell fate determining programs such as cell differentiation and growth arrest (Yu et al., 2010).

**FIGURE 6:**
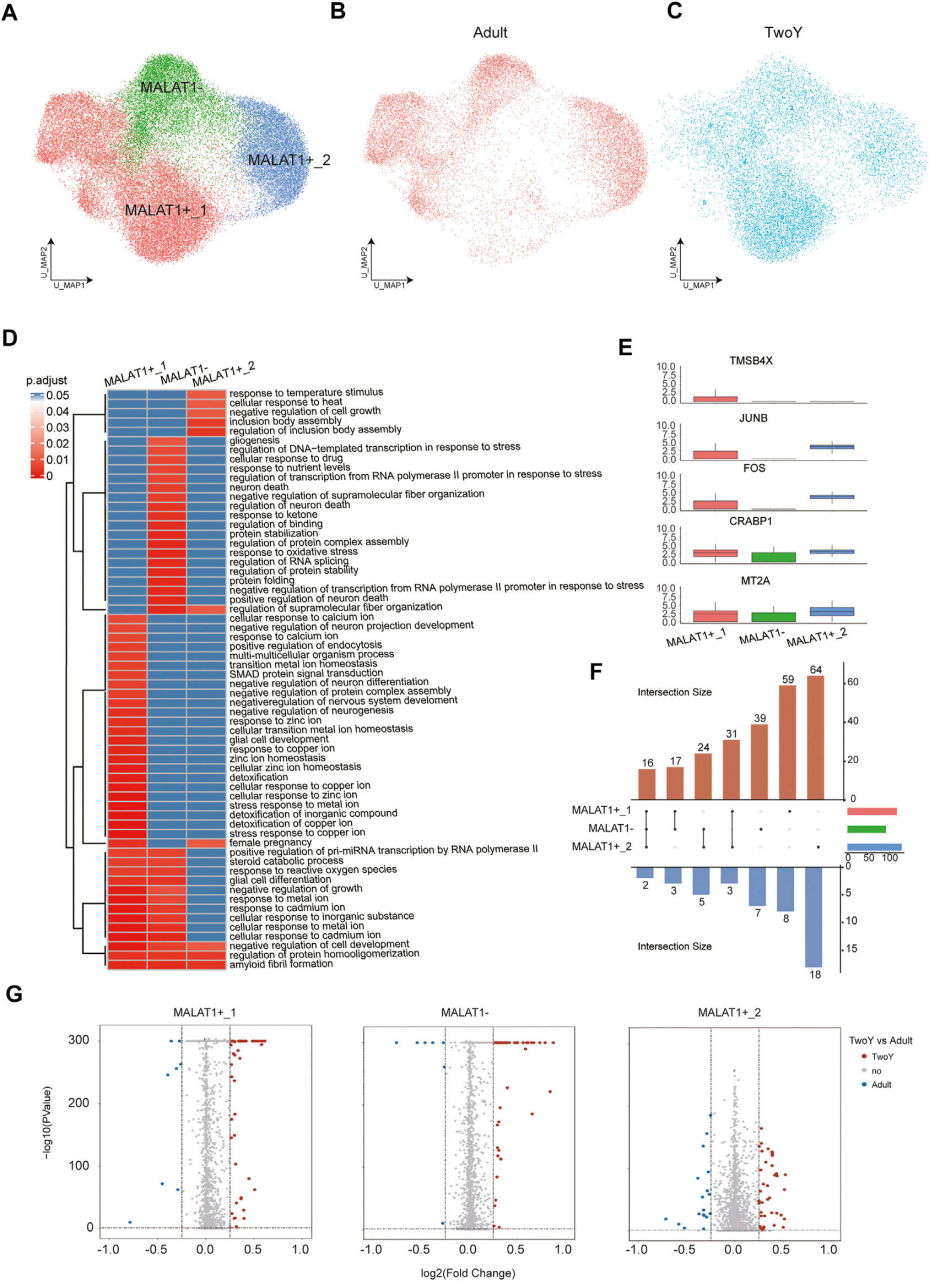
Comparison with reference data from adult rod PRs. **(A)** A UMAP map of reference adult data and TwoY data among rod PRs colored by clusters. **(B)** A UMAP map of reference adult data within rod PRs. **(C)** A UMAP map of TwoY data within rod PRs. **(D)** Heatmap shows the enrichment level of GO terms in each cell type. **(E)** Boxplots show the expression patterns of differentially expressed genes in each cell subtype. **(F)** An Upset plot enables visualization of the number of up-regulated and down-regulated genes in each cell subtype (TwoY vs. adult). The cell subtypes connected by the straight line in the middle refer to the subpopulations with the same differentially expressed genes. The corresponding blue bars below refer to the number of co-owned down-regulated genes, and red bars above refer to the number of co-owned up-regulated genes. The columns on the right represent the total number of differential genes between TwoY and adult in each subtype. **(G)** Volcano plot showing the differentially expressed genes in each cell types between the retinas of adults and young children. Genes are plotted as log2 fold change versus the −log10 of the adjusted *p*-value. Genes in red denote upregulation in the retina of young children, while blue denotes upregulation in adult retina. Insignificant genes are colored in grey. Thresholds are shown as dashed lines.

MALAT1 is a long-chain noncoding RNA that plays a role in retinal homeostasis and disease. Lukowski et al. have found that the differential expression of MALAT1 can be used to identify rod cells in different cellular states, namely, healthy or degenerating rod PRs. It has indicated that the rod subpopulations with low MALAT1 expression represent putative early degenerating rod PRs, and MALAT1 can be considered as a potential target to enhance the survival of rod PRs (Lukowski et al., 2019). In our study, we employed the same analytical approach to investigate the heterogeneity of rod PRs. In our rod PRs dataset, unsupervised reclustering produced 3 subpopulations, namely, C0, C1, and C2. C0 was annotated as MALAT1-, showing the lowest expression of MALAT1. C1 and C2, with higher expression of MALAT, were labeled MALAT1+_1 and MALAT1+_2, respectively (Figures 6A–C). These three cell subpopulations reflected the different stages of degeneration in rod PRs. MALAT1- might potentially represent the early degeneration stage, while MALAT1+_1 and MALAT1+_2 referred to relatively healthy Rod subpopulations. Also, our results showed that the cell numbers of the MALAT1- subpopulation were significantly reduced in young children compared with adults. It suggested that the degeneration of rod PRs in young children was insignificant, relative to adults. We further explored the function of DEGs by functional enrichment analysis. The GO terms of the three cell types were conducive to further understanding the three subpopulations (Figure 6D). The analysis results showed that compared with MALAT1+_1 and MALAT1+_2, MALAT1- were more related to neuron death. More significantly, we found that the genes related to cell proliferation and cell survival (*TMSB4X*, *JUNB*, *FOS*, *CRABP1*, and *MT2A*) were significantly overexpressed in MALAT1+_1 or MALAT1+_2 subtypes (Figure 6E). Volcano plots showed that the upregulated/downregulated genes in MALAT1+_1, MALAT1+_2, and MALAT1- were obvious (Figure 6F). In addition, we visualized the dataset of DEGs, showing the number of elements in each cell type (Figure 6G). MALAT1+_2 cells were the most numerous, and the number of upregulated genes was greater than the number of downregulated genes in both related cell types and independent cell types.

**FIGURE 7:**
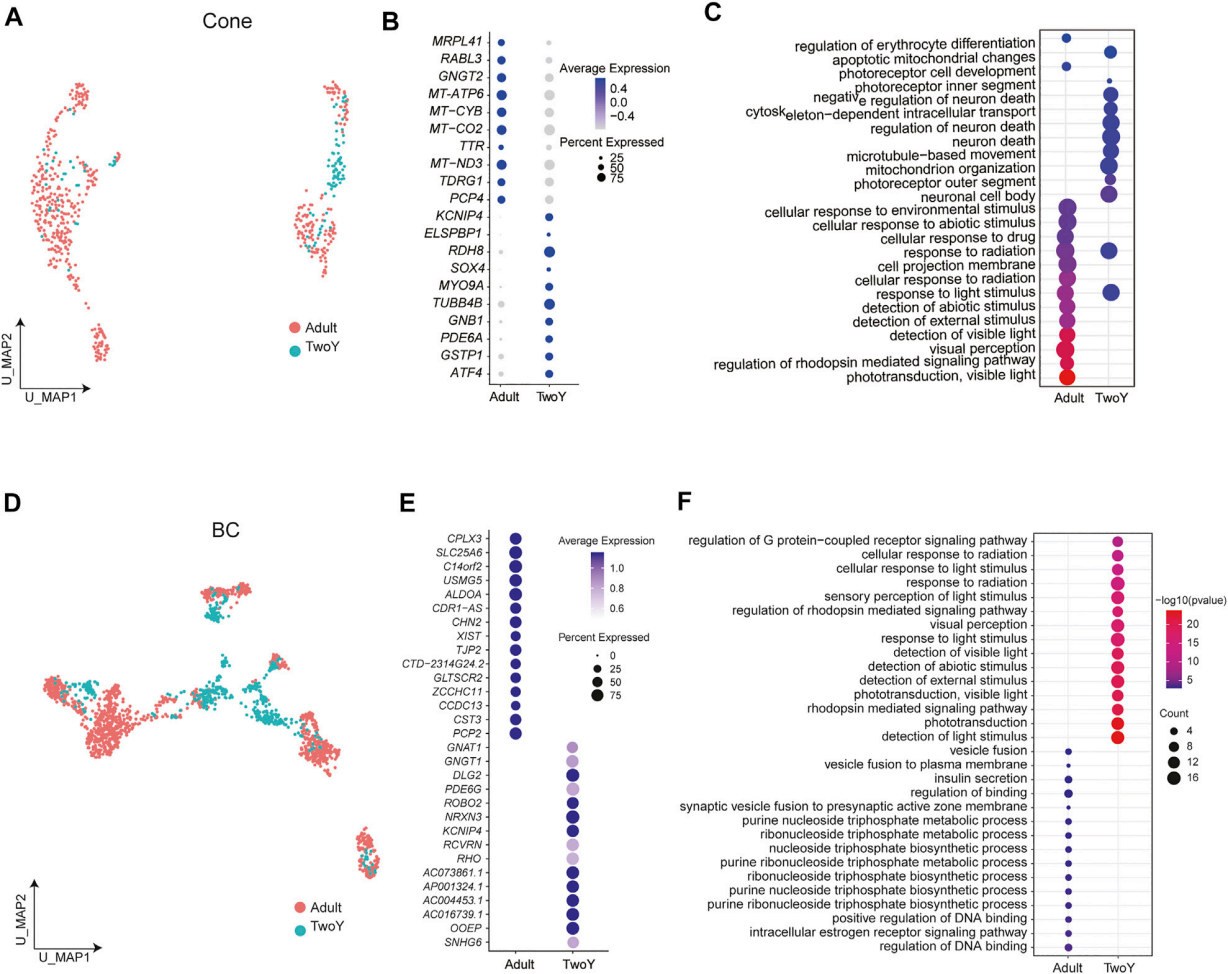
Comparison of BCs and cone PRs between the retinas of adults and young children. **(A)** A UMAP map of reference adult data and TwoY data among cone PRs, separated by data source. **(B)** Different expression patterns of cone PR markers in adults and young children. Dot size represents the percentage of cells expressing corresponding genes and color represents the expression levels. **(C)** A Dotplot shows functional enrichment of genes with significant differential expression between the cone PRs of adults and young children. **(D)** A UMAP map of reference adult data and TwoY data within BCs, separated by data source. **(E)** Different expression patterns of BC markers in adults and young children. Dot size represents the percentage of cells expressing corresponding genes and color represents the expression levels. **(F)** A Dotplot shows functional enrichment of genes with significant differential expression between the BCs of adults and young children.

Finally, cone PRs and BCs in adults and young children were further analyzed, revealing the specific expression of genes (Figures 7A,B,D,E). For instance, *KCNIP4*, *RDH8*, *MYO9A*, and *TUBB4B* were specifically expressed in the cone PRs of young children, while *MRPL41*, *RABL3*, and *GNGT2* were highly expressed in adults (Figure 7B). In addition, compared with the cone PRs of adults, GO term enrichment analysis revealed that those of young children were associated with photoreceptor differentiation, while adults showed more mature cone PRs properties, such as visual perception (Figure 7C). For BCs, *GNAT1*, *ROBO2*, *NRXN3*, and *DLG2* were highly expressed in young children, while *CPLX3*, *SLC25A6*, and *PCP2* were differentially expressed in adults (Figure 7E). As shown in Figure 7F, the GO terms of BCs in young children were enriched in the sensory perception of light stimulus, whereas those of adults were involved in the synaptic vesicle fusion.

## Discussion

This complex intrinsic composition of retinal cells highlights the necessity of single-cell technologies for the detailed dissection of transcriptome signatures. Although several papers regarding fetal, postnatal and adult retinas have been published, no group has yet published an atlas of the retina from the infant and young child. The maturation and aging of the retina are of significant value in the study of its development and pathology. We believe that the data resources and insights obtained in our study could be a very useful addition to this field.

Here, we report the first single-cell atlas of the human retina at different developmental stages. Our dataset consisted of 194,967 cells and nuclei, achieving an unprecedentedly large scale for human retina research. We also identified microglia, which are considered the resident immune cells in the human retina, and found that microglia showed putative interactions primarily with rod PRs, BCs, and MGs through ligand-receptor communication network analysis, implying that rod PRs, BCs, and MGs might play an important role in immune homeostasis.

We next sought to identify subtypes and subpopulations of PRs and BCs through reclustering. Among cone PRs, we identified S-cones, M-cones, and L-cones. For BCs, reclustering resulted in 11 subpopulations; 5 of these subpopulations were classified as OFF BCs, while the 3 subpopulations were thought to be ON BCs according to the expression of *GRIK1* and *GRM6*. We further described the different properties of each subpopulation by assessing the expression pattern of function-related ion channels. The above results suggested that functional subtypes of BCs might be identified in early human retinas from snRNA-seq data, further experimental verification needs to be conducted.

The distinctions between the retinas of young children and adults were also illuminated in this study, with a focus on the differences in the function and gene expression of retinal cells at different stages of development and the determination of specific cell subtypes with characteristic genes. First, our analysis data revealed that the primary function of MGs in the retina of young children was sensory system development and visual system development, while those of adults were mainly involved in transmembrane transport and ion transport. Based on the differences in MALAT1 expression patterns, rod PRs were divided into three cell subtypes, namely, MALAT1+_1 and MALAT1+_2, with higher MALAT1 expression, and MALAT1-, with lower MALAT1 expression. The differences in gene expression and cellular function among these three subclusters were confirmed through system analysis. This enabled us to form a deeper understanding of the cell characteristics of these three subtypes. In general, the gene expression patterns of PRs and BCs in the retina of young children were significantly different from those in the adult retina. Our results revealed significant differences in cell state between the retinas of young children and adults.

IRDs are a group of retinal disorders caused by inherited gene mutations and can result in vision loss or blindness (Edwards et al., 2011; Thomas et al., 2014). Currently, disease diagnosis and therapies are hampered by a limited understanding of the human retina at the single-cell level. In our retinal disease analysis, S-cones manifested nonsignificant enrichment of all common retinal diseases included in our study, while M-cones were found to be associated with CRD and RP. Taken together, our findings narrowed down the disease-associated cell subtypes and subpopulations and their corresponding biomarkers, which might not only shed light on the molecular mechanisms underlying IRDs but also provide new insights for the discovery of novel drug targets and the development of gene therapies for IRDs.

## Conclusion

In conclusion, we constructed the first transcriptome profiles of the human retina from infants and young children at single-cell resolution, which provided valuable clues regarding the postnatal development of the human retina. The subtypes of retinal cells with specifically expressed genes were also identified. In addition, we linked IRDs to certain cell subpopulations along with corresponding molecular markers. By evaluating the similarities and differences between the retinas of adults and young children, we reported differentially expressed genes and various patterns of functional enrichment among different stages of development in the human retina.

## Data Availability

The data presented in the study are deposited in the Figshare repository, accession number doi.org/10.6084/m9.figshare.19028594.
