# Supplementary figures and images for: Single-Cell RNA-Seq Reveals the Cellular Diversity and Developmental Characteristics of the Retinas of an Infant and a Young Child
Source: Front Cell Dev Biol. 2022 Mar 21;10:803466. doi: 10.3389/fcell.2022.803466 (PMC8979067; doi:10.3389/fcell.2022.803466)

Supplementary Figure S2

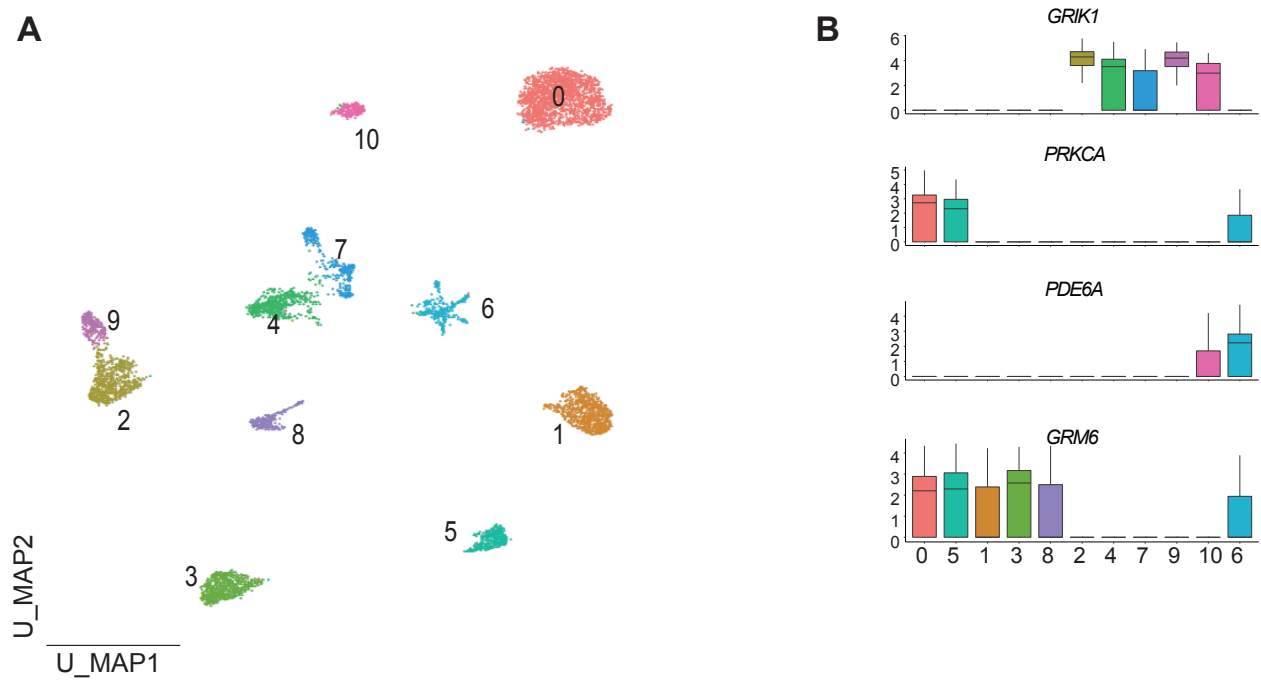

Supplement: Supplementary file 1 [file DataSheet2.PDF]
