# Supplementary material for: Single-Cell RNA-Seq Reveals the Cellular Diversity and Developmental Characteristics of the Retinas of an Infant and a Young Child
Source: Front Cell Dev Biol. 2022 Mar 21;10:803466. doi: 10.3389/fcell.2022.803466 (PMC8979067; doi:10.3389/fcell.2022.803466)

Supplementary Figure S1

A

Library	TenM4	TenM5	TenMZ	TwoY6	TwoY8	TwoYZJ
Technique	snRNA	snRNA	snRNA	snRNA	snRNA	scRNA
Age	10 Months	10 Months	10 Months	2 Years	2 Years	2 Years
Mean reads per cell	128,753	58,402	38,802	8001	39,066	22,701
Median genes per cell	461	381	426	316	680	513
Sequencing saturation	90.80%	89.10%	81.10%	85.1%	85.10%	82.70%

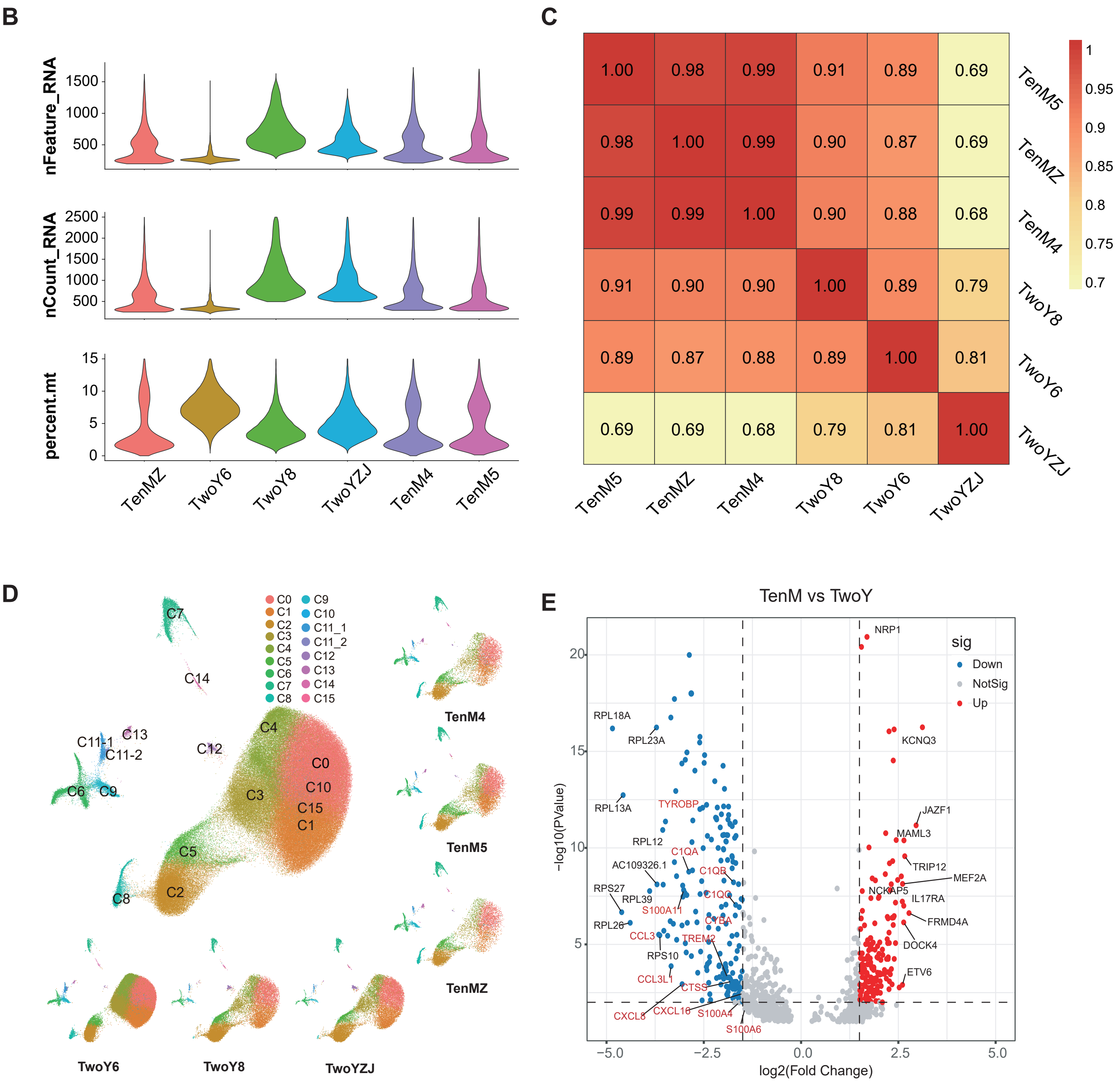

Supplement: Supplementary file 4 [file DataSheet1.PDF]
